# Supplementary material for: Decreased lung function is associated with elevated ferritin but not iron or transferrin saturation in 42,927 healthy Korean men: A cross-sectional study
Source: PLoS One. 2020 Apr 2;15(4):e0231057. doi: 10.1371/journal.pone.0231057 (PMC7117746; doi:10.1371/journal.pone.0231057)
Supplement: Attachment — Submitted filename: Response to Reviewers.docx [file pone.0231057.s001.docx]

**Manuscript ID : PONE-D-19-26396**

**Title: Decreased lung function is associated with elevated ferritin, but not iron or transferrin saturation in 42,927 healthy Korean men: a cross-sectional study**

Thank you for the constructive comments and criticisms of our manuscript named above. In response to the reviewer’s suggestions and questions, we have revised the manuscript as suggested. We have responded to the reviewer’s comments in a point-by-point manner below. We hope that this work is now suitable for publication and look forward to your response.

PS) As we response to Journal Requirements, we must make changes to our original Data Availability statement. Would you please let us allow to state bellows instead of original statement?

Due to ethical restrictions imposed by the Institutional Review Board of Kangbuk Samsung Hospital, the patient

data are not available for distribution outside of the Kangbuk Samsung Hospital. For additional information, please contact JiinAhn (Jiin57.ahn@samsung.com) or the Institutional Review Board of Kangbuk Samsung Hospital (contact information below). - Address: 29 Saemunan-ro, Jongno-Gu, Seoul, Korea (03181) - E-mail: irb. kbsmc@samsung.com - Telephone: 82-2-2001-1943, 1945 - Fax: 82-2-2001-1946.

Lastly, we wish you to allow to add financial support as bellows;

**Financial Support:** The 2019 scientific promotion program funded by Jeju National University.

We apologize for our mistakes and carelessness.

With best wishes

Jae-Uk Song, MD, PhD,

Division of Pulmonary and Critical Care Medicine, Department of Internal Medicine, Kangbuk Samsung Hospital, Sungkyunkwan University School of Medicine,

29 Saemunan-ro, Jongno-gu, Seoul 03181, Republic of Korea

Phone: +82-2-2001-1597

Fax: +82-2-2001-1596

E-mail: [khfever76@gmail.com](mailto:khfever76@gmail.com)

**Point-by-point responses to reviewer’s comments**

**TO THE COMMENTS OF THE JOURNAL REQUIREMENTs**

C1

We noticed you have some minor occurrence(s) of overlapping text with the following previous publication(s), which needs to be addressed:
<https://doi.org/10.1371/journal.pone.0208736>
<https://doi.org/10.1111/resp.13370>
<https://doi.org/10.1080/14397595.2017.1285981>

In your revision ensure you cite all your sources (including your own works), and quote or rephrase any duplicated text outside the Methods section. Further consideration is dependent on these concerns being addressed.

C1

Thank you for your kind advice. Actually we have some overlapping texts with our own works. We concisely condense a method section by omitting duplicated texts and citing the sources. And, we modified most part of our original manuscript according to reviewer’s comments. You can find highlights changes in 'Revised Manuscript with Track Changes'

**(from line 82 on page 4 to line 102 on page 5);**

This study began with data from 189,154 individuals who underwent a comprehensive health examination at Kangbuk Samsung Hospital Health Screening Centers in 2015. Initially, we extracted 188,596 participants with a recorded serum ferritin level and spirometry data. We excluded 80,003 participants who had a self-reported history of disease or were currently receiving medication for any disease. Specific details of comorbidities were unavailable because the medical history questionnaire required only yes/no responses. We also excluded 11,942 subjects with missing medical history data or data about smoking habits or alcohol consumption. Because some subjects had more than more than one exclusion criterion, 96,651 subjects were ultimately eligible for initial inclusion in the study. Of these, we excluded women because the proportion of women with hyperferritinemia, defined as > 200 mg/dL, was only 0.9%, leaving too few women for us to obtain statistically valid results. Thus, we decided to analyze the data from 47,981 men (Figure 1).

🡪

This study began with data from 189,154 individuals who underwent a comprehensive health examination at Kangbuk Samsung Hospital Health Screening Centers in 2015. Initially, we extracted 188,596 participants with a recorded serum ferritin level and spirometry data. We excluded 85,455 participants who had a ventilation disorder on the basis of spirometric results [1] and self-reported history of disease or were currently receiving medication for any disease to 1) test our hypothesis with robustness, 2) yield more meaningful results, and 3) minimize potential confounders. Specific details of comorbidities were unavailable because the medical history questionnaire required only yes/no responses. We also excluded 11,942 subjects with missing medical history data or data about smoking habits or alcohol consumption. Because some subjects had more than more than one exclusion criterion, 91,199 subjects were ultimately eligible for initial inclusion in the study. The reference range for serum ferritin is usually from 30 to 300 ng/mL in men and from 15 to 200 ng/mL in women. These ranges seem to vary across reference populations and according to age and sex, which are the most important determinants of serum ferritin [2]. There has been no consensus on criteria for hyperferritinemia. Our cohort consisted of mostly middle aged Asians with higher socioeconomic and education levels and urban habitation with the highest annual PM concentrations in the world. Thus, these characteristics can contribute to more increase in the amount of mobilized iron especially in men than women in our cohort [3-9]. The large difference in serum ferritin level between men and women can contribute to much higher lifetime iron stores in men than in women. Therefore, the prevalence of hyperferrtinemia can be markedly different between men and women. Accordingly, we excluded women due to the small proportion (451, 0.9%) with hyperferritinemia, defined as > 200 ng/mL, leaving too few women for us to obtain statistically valid results. Thus, we decided to analyze the data from 42,927 men (Fig. 1)

**(from line 119 on page 5 to line 129 on page 6 );**

Physical characteristics and serum biochemical parameters were measured by trained nurses. Height and weight were determined using automated instruments, with individuals shoeless and wearing a lightweight hospital gown. Height was measured to the nearest 1 mm using a stadiometer with the participant standing barefoot. Weight was measured to the nearest 0.1 kg on a bioimpedance analyzer (InBody 3.0 and Inbody 720, Biospace Co., Seoul, Korea) that was validated for the reproducibility and accuracy of body composition measurements[10]. Body mass index was calculated as weight in kg divided by height in m^2^. Obesity was defined as a BMI ≥25 kg/m^2^, which is the proposed cut-off for Asian populations [11]. Blood pressure was measured with a standard sphygmomanometer following at least 5 minutes of seated rest. Measurements were performed twice at 5-min intervals and averaged for analysis. Mean BP (MBP) was calculated as diastolic BP + 0.33 × (systolic BP − diastolic BP).

Blood samples were collected from the antecubital vein after at least a 10-h fast. The methods used to measure serum levels of glucose, lipid profiles, liver enzymes, creatinine, insulin, and high-sensitivity C-reactive protein (hsCRP) have been described previously [12, 13]. Insulin resistance was assessed using the homeostasis model assessment of insulin resistance (HOMA-IR) equation: fasting blood insulin (μU/ml) × fasting blood glucose (mmol/l)/22.5 [14]. The serum iron concentrations were measured using an automatic chemistry analyzer (Cobas 8000 c702; Roche Diagnostics, Tokyo, Japan) and a colorimetric assay. The serum ferritin was determined using an electrochemiluminescence immunoassay (Cobas 8000 e602; Roche Diagnostics) based on the sandwich principle. We defined subjects with ferritin > 300 mg/dL, TSAT > 50%, and iron > 175 μg/dL as having hyperferritinemia, high TSAT, and high iron, respectively [2, 15].The Laboratory Medicine Department at Kangbuk Samsung Hospital has been accredited and participates annually in inspections and surveys by the Korean Association of Quality Assurance for Clinical Laboratories.

🡪

Physical characteristics and serum biochemical parameters were measured by trained nurses, as previously reported [16, 17]. Obesity was defined as Body mass index (BMI) ≥25 kg/m^2^ [11]. Insulin resistance was assessed using the homeostasis model assessment of insulin resistance (HOMA-IR) equation: fasting blood insulin (μU/ml) × fasting blood glucose (mmol/l)/22.5 [14]. Serum iron concentration was measured using an automatic chemistry analyzer (Cobas 8000 c702; Roche Diagnostics, Tokyo, Japan) and a colorimetric assay. The serum ferritin was determined using an electrochemiluminescence immunoassay (Cobas 8000 e602; Roche Diagnostics) based on the sandwich principle. We defined subjects with ferritin > 300 ng/mL, TSAT > 50%, and iron > 175 μg/dL as having hyperferritinemia, high TSAT, and high iron, respectively [2, 15]. The Laboratory Medicine Department at Kangbuk Samsung Hospital has been accredited and participates annually in inspections and surveys by the Korean Association of Quality Assurance for Clinical Laboratories.

C2

We note that you have stated that you will provide repository information for your data at acceptance. Should your manuscript be accepted for publication, we will hold it until you provide the relevant accession numbers or DOIs necessary to access your data. If you wish to make changes to your Data Availability statement, please describe these changes in your cover letter and we will update your Data Availability statement to reflect the information you provide.

R2

We really appreciate for your consideration. We apologize for our mistakes and carelessness. Due to ethical restrictions imposed by the Institutional Review Board of Kangbuk Samsung Hospital, the patient

data are not available for distribution outside of the Kangbuk Samsung Hospital. A couple year ago, a doctor (who used to work in our hospital) was charged for leaking our health screening data because he shared our data with other doctors without legal permission. After that, we only approach heath screening data only inside computer network (called “Cohort VDI system”) in our hospital. So, it is impossible to leak our data to the outsiders. This is way beyond my jurisdiction. So, we shouldn’t have stated approval for providing our data. We again apologize for our mistakes to state that we will provide our data.

Therefore, we must make changes to our original Data Availability statement. Would you please let us allow to state bellows instead of original statement ? Once again, we really appreciate to realize our mistake and give a chance to correct.

Due to ethical restrictions imposed by the Institutional Review Board of Kangbuk Samsung Hospital, the patient

data are not available for distribution outside of the Kangbuk Samsung Hospital. For additional information, please contact JiinAhn (Jiin57.ahn@samsung.com) or the Institutional Review Board of Kangbuk Samsung Hospital (contact information below). - Address: 29 Saemunan-ro, Jongno-Gu, Seoul, Korea (03181) - E-mail: irb. kbsmc@samsung.com - Telephone: 82-2-2001-1943, 1945 - Fax: 82-2-2001-1946.

Lastly, we wish you to allow to add financial support as bellows;

**Financial Support:** The 2019 scientific promotion program funded by Jeju National University.

**TO THE COMMENTS OF THE REVIEWER 1.**

C1.

Authors in this study conducted a cohort study of 47,981 healthy Korean men, and found hyperferritinemia was associated with decreased lung function in healthy Korean men, whereas iron and TSAT were not. In general, this finding is interesting and useful, even though longitudinal follow-up studies and prospective interventional studies are needed to validate it. It better to discuss the relationship between iron and oxygen concentration in discussion, as it was mentioned previously that different iron need under different oxygen concentration. (Cancer Lett. 2019 Nov 1;464:56-61.)

R1.

We do appreciate the reviewer’s comment. And we really appreciate the helpful reference. As reviewer pointed out, tissue oxygen concentration could affect iron level[18]. At a higher oxygen concentration within tissue, degradation of iron-sulfur clusters, which are essential for the activity of diverse proteins, could cause insufficient iron–sulfur cluster maintenance. Thereafter, it activates the iron-starvation response and ferroptosis[19]. As a rule, tissue oxygenation is the resultant of the oxygen utilization which is mostly determined by the arterial O2 concentration, and the arterial O2 concentration is mostly determined by the shape of the O2 dissociation curve[20]. Specifically, the oxyhemoglobin dissociation curve relates oxygen saturation (SO_2_) and partial pressure of oxygen in the blood (PaO_2_). Therefore, PaO_2_ or SO_2_ can affect iron metabolism. Unfortunately, present study was not hospital based. Lack of measurements of PaO_2_ or SO_2_ led us not to evaluate relationship between iron and oxygen concentration in current study.

It is only fair that we acknowledge another limitation of this study, because this may not now be possible.

As you recommended, we added sentences in the manuscripts as follows;

**(from line 356 to line 361 on page 26);**

Finally, this study was not hospital-based. Lack of data on genetic and environmental variations that could have influenced our results, including daily dietary iron, is also a potential limitation of this study.

🡪

Finally, this study was not hospital-based. Lack of data on genetic and environmental variations that could have influenced our results, including daily dietary iron, is another potential limitation of this study. Additionally, lack of measurements of oxygen saturation and partial pressure of oxygen in the blood (PaO_2_), which could affect iron metabolism [18], are also potential limitations, although we excluded those with any known evidence of clinical medical diseases including lung disease.

**TO THE COMMENTS OF THE REVIEWER 2.**

This study looks at the relationship of ferritin, iron and iron saturation in relationship to lung function and other parameters of systemic inflammation. The study population is ~47000 men representing ~25% of a large screening program from a single Korean hospital, after excluding all with history of any disease or taking any medication. Unlike other, population based, studies of this question which have looked at correlations to the spectrum of ferritin levels, the authors have chosen to focus on the likelihood of a ferritin level above the upper limit of normal (ULN). Women were excluded because <1% exceeded the chosen ULN. The results in relationship to lung function appear discordant with prior studies and potential reasons for this are discussed.

**Major comments:**

C1.

The study premise is that elevated ferritin is associated with systemic inflammation and pulmonary injury and thus may be associated with decreased lung function, in what would presumably be a pathologic process. However, the study population excluded those with any known evidence of lung disease so the lung function data available are nearly all within the normal range of a healthy population where most of the variation above and below the predicted value is considered to be random rather than pathologic. Certainly, some individuals with early disease processes will have low normal values, but this choice would seem to create a low signal to noise situation. The rationale for the choice to exclude even mild airflow limitation from the study population should be explained.

R1

Thank you for your reasonable comments. The reviewer makes a very important point.

As you pointed out, elevated ferritin is associated oxidative stress and inflammatory processes, which disrupt normal cell function. This process could be a pathologic process. However, inhaled air contains a variety of potentially harmful particulate matter (PM). These smaller PM can pass freely across biological membranes, such as the lung, to become systemically dispersed into cells, where they disrupt normal cell function. By the way, air pollution particulates contain transition metals, such as iron (usually most abundant) which can be mobilized from the PM inside human lung epithelial cells and disrupt iron homeostasis both in the lung and systemically[9, 21].

Inappropriate levels of iron inside lung epithelial cells can result in ferritin release from damaged cell which could result in elevated serum ferritin concentration, losing most of its iron and leaving free iron[22]. This bioavailable iron is responsible for reactive oxygen species (ROS) and IL-8 induction (inflammatory mediator) [9]. Therefore, airway epithelial cells that line the conducting airway could be exposed to reactive oxygen species (ROS) and inflammatory mediators even in health subjects. Wherein there can be possibility for the damaging effects of iron on the respiratory health of healthy subjects[23], who especially live or work in urban area (Seoul metropolitan areas have higher outdoor air pollution burden[3], as a consequence of rapid regional urbanization and industrialization), even under normal physiological conditions not pathological diseases.

In addition, recent studies have continuously shown that pulmonary[24] and cardio-metabolic diseases [12, 13, 25-28] are a recognized complication of excess iron accumulation. And, these patients are more prone to poor lung function [24, 29]. So, we believe that inclusion patients with previous clinical diseases could distort the magnitude of an association between lung function and ferritin.

For that reason, we only included healthy subjects in order to 1) test our hypothesis with robustness, 2) yield more meaningful results and 3) to minimize potential confounder. However, the true incidence of pulmonary and cardio-metabolic diseases could been underestimated in our study due to the questionnaire-based collection of medical histories. And, some individuals with subclinical disease processes could also be included, although we additionally excluded the subjects with abnormal ventilation disorder such as obstructive pattern, restrictive pattern and mixed pattern which have been associated with higher ferritin level[24]. As reviewer concerned, these may seem to be significant, because the relationship between subclinical pulmonary and cardio-metabolic diseases can contribute to a decline in lung function, especially among individuals with abnormal ferritin levels.

So, we add some sentences in introduction section to present the putative rationale behind the association between hyper-ferritin and lung function in healthy subjects. We also exclude the subjects with ventilation disorder on the basis of spirometric results. We hope this exclusion could be an answer to your second question in minor comments. Nonetheless, similar results were observed in the subjects only with normal lung function (n= 42,927). And, we clarify why we only select healthy subjects in this study in method section as follows;

**(from line 52 to line 65 on page 3);**

However, ferritin can become elevated in the presence of inflammation irrespective of iron status, and inflammation can contribute to various cardio-metabolic diseases [22]

Decreased lung function is also associated with systemic inflammation [30] and an increased risk of insulin resistance, diabetes, and cardiovascular disease[29]. In addition, elevated serum ferritin is associated with an increased risk of pulmonary injury [22], suggesting that decreased lung function might be associated with an elevated serum ferritin level.

🡪

Ferritin also can become elevated in the presence of oxidative stress and inflammation irrespective of iron status and can contribute to various clinical diseases, especially pulmonary and cardio-metabolic diseases [22, 24]. Moreover, decreased lung function is associated with oxidative stress and systemic inflammation [30] and increased risk of insulin resistance, diabetes, and cardiovascular disease [29]. This suggests that decreased lung function could be associated with elevated serum ferritin level in pathological conditions.

Even in healthy subjects, airway epithelial cells could be exposed to oxidative stress and inflammation because of ambient air pollution aerosols, which recently have increased rapidly as a consequence of regional urbanization and industrialization [23]. Air pollution particulate matter (PM) contains transition metals such as iron (usually most abundant), which can be mobilized from the PM to lung epithelial cells and disrupt iron homeostasis both in the lung and systemically [9, 21]. Iron overabundance relative to metabolic needs inside lung epithelial cells can result in ferritin release from damaged cells, which could result in elevated serum ferritin concentration and loss of lung function under normal physiological conditions [9, 21, 23].

**(from line 66 on page 3 to line 78 on page 4);**

However, epidemiological evidence to support that hypothesis is lacking. To date, only four reports have investigated a potential association between ferritin and lung function [31-34]. Two of them found a positive association between serum ferritin and lung function [31, 32], and the other two found no association [33, 34]. Thus, the exact nature of the relationship between ferritin and lung function, if any exists, remains unclear. It also remains unclear whether the previous results reflect a relationship between lung function and a recognized marker of iron status or an epiphenomenon in which an increased ferritin level reflects overall inflammation. Therefore, we investigated the relationships between lung function and ferritin and lung function and other biomarkers of iron metabolism, including transferrin saturation (TSAT), in healthy Korean men.

🡪

Nevertheless, epidemiological evidence to support that hypothesis is scarce. To date, only four reports have investigated a potential association between ferritin and lung function [31-34]; two of them found a positive association [31, 32], and the other two found no association [33, 34]. However, these previous studies were not conducted only in healthy subjects. Recent studies have continuously shown that pulmonary[24] and cardio-metabolic diseases [12, 13, 25-28] are a recognized complication of excess iron accumulation, and such patients are prone to poor lung function [24, 29]. This suggests that inclusion of patients with clinical disease could distort the magnitude of association between lung function and ferritin. Thus, the exact nature of the relationship between ferritin and lung function, if one exists, remains unclear. It also remains unclear whether the previous results reflect a relationship between lung function and a recognized marker of iron status or an epiphenomenon in which an increased ferritin level reflects overall inflammation. Therefore, we investigated the relationships between lung function and ferritin and lung function and other biomarkers of iron metabolism, including transferrin saturation (TSAT), in healthy Korean men.

**(from line 32 on page 2);**

We conducted a cohort study of 47,981 healthy Korean men (mean age: 38.9 years).

🡪

We conducted a cohort study of 42,927 healthy Korean men (mean age: 38.6 years).

**(from line 84 to line 87 on page 4);**

We excluded 80,003 participants who had a self-reported history of disease or were currently receiving medication for any disease.

🡪

We excluded 85,455 participants who had a ventilation disorder on the basis of spirometric results [1] and self-reported history of disease or were currently receiving medication for any disease to 1) test our hypothesis with robustness, 2) yield more meaningful results, and 3) minimize potential confounders.

**(from line 273 to line 282 on page 23);**

Additionally, we adjusted for many relevant confounders associated with ferritin and lung function and examined only males. After fully adjusting for potential confounders, we found a robust negative association between hyperferritinemia and lung function in healthy men.

🡪

Additionally, we adjusted for many relevant confounders associated with ferritin and lung function and examined only males. After fully adjusting for potential confounders, we found a robust negative association between hyperferritinemia and lung function in healthy men. These results indicate that healthy subjects with hyperferritinemia may have early perturbations of lung function. This finding is important. Reduced lung function is a marker of an individual’s increased susceptibility to COPD and a major risk factor for cardiovascular morbidity and mortality, which are potentially preventable diseases with significant health and economic impacts worldwide [35]. Further, reduced lung function is a powerful predictor of mortality [36]. Therefore, the current study is important, given the projected growing public health impact of lung function and outdoor air pollution [23].

**(from line 339 on page 25 to line 342 on page 26);**

Another strength is that we conducted analyses adjusting for various confounders that affect lung function and ferritin.

🡪

Another strength is that we conducted analyses adjusting for various confounders that affect lung function and ferritin. This gave us sufficient statistical power and could be more relevant to healthy population with normal lung functions.

we added another limitation of current study in the manuscripts as follows;

**(from line 350 to line 356 on page 26);**

Fourth, the true incidence of pulmonary and cardio-metabolic diseases could been underestimated in our study due to the questionnaire-based collection of medical histories. And some individuals with subclinical disease processes could also be included, although we were trying to exclude subjects with overt medical diseases and ventilation pattern. This may seem to be significant, because the relationship between subclinical pulmonary and cardio-metabolic diseases can contribute to a decline in lung function, especially among individuals with abnormal ferritin levels.

C2

Since women are known to have different ferritin levels and behaviors (eg, with age) than men, it is unfortunate that this population was excluded by the choice to consider only hyper-ferritinemia rather than a median, mean or correlation to the range of values. Since this was essentially a normal, disease free population one might expect 2.5% to be above the ULN (assuming a 95% range and false positives at both ends) so the finding of <1% suggest the ULN chosen for women may be too high. Similarly, the finding of nearly 20% of these healthy men to be above the ULN (rather than the expected 2.5 %) needs some discussion, starting with the source and derivation of these cut points (general population or “healthy” pop, smokers excluded or not, appropriate to Korean ethnicity?). An alternative analysis of “hyper” ferritin, that would allow inclusion of women, would be to divide the groups at the 80th %iles for men and women of the study population.

R2

We do appreciate the reviewer’s comment. We fully understand reviewer’s concern.

Actually, the reference levels for serum ferritin are wide, but usually from 30 to 300 ng/mL in men and from 15 to 200 ng/mL in women. This variability seems to vary across laboratories due to differences in analytical techniques, reference populations in addition to age and sex which are most important determinants of serum ferritin.[2]

Serum ferritin level of our cohort was much higher in men (median: 199 ng/mL, mean: 220 ng/mL) not women (mean 49.6 ng/mL, median: 38.9 ng/mL), and there are greater gaps in serum ferritin level between men and women, compared to previous studies [31-34], although our cohort was consisted of only healthy subjects with normal lung function.

The higher ferritin level in men but comparable level in women have some possible explanation.

First, gender differences concerning the association between serum 25 (OH)D level and ferritin might be considered [5]. Serum 25(OH)D level was inversely associated with ferritin level in men, and the opposite association in premenopausal women, and was not associated with the serum ferritin levels in postmenopausal women [5], because of sex hormones[37-40]. In South Korea, the general prevalence of vitamin D deficiency (75.2%-82.5%)[4] is significantly much higher than the global prevalence (around 50% of the population)[8].

Interestingly, predictors for vitamin D deficiency in the Korean population included a younger age group and living in an urban area with indoor occupations [7]. Additionally, ethnic populations can impact serum ferritin concentrations. Asians had the highest geometric mean levels of serum ferritin, compared to white, black, and American Indian [41]. The majority of our subjects were middle aged city dwellers with in-door occupation and a higher socioeconomic level. Thus, this is related to higher ferritin level in our men but those in women was comparable to previous studies [31-34], even under normal physiological conditions.

Second, outdoor air pollution is getting worse globally as urban populations and car numbers increase [3, 23]. In contrast to the pollution encountered in rural setting or low- and middle income countries, air pollution in urban area (especially in Seoul) comprises smaller particles with a mass median diameter of 2.5μm (PM2.5), originating from transport. Annual PM2.5 concentrations in Seoul metropolitan areas was much higher in America and also higher than other areas even in South Korea [3]. The amount of bioavailable iron released from air pollution particulates is dependent on the particle size, showing an increase in the amount of iron that can be mobilized with decreasing particle size[9]. Additionally, men might be much more susceptible to iron induced free radical injuries than women [42]. It might be contributed to higher level of ferritin in men, not women beyond regular blood loss through menstruation in premenopausal women, than those in previous studies. [31-34]

Wherein we assumed that the characteristics of our cohort may be related to the reviewer’s concern, especially in men. The characteristics of our cohort consisted of mostly middle aged Asians with a higher socioeconomic and education level and urban habitation with the highest annual PM2.5 concentrations in the world can contribute to more increase in the amount of iron that can be mobilized. So, the prevalence of hyperferritinemia (defined as > 300 ng/mL in men) can be higher than expected. And, we selected this level, based on the usual upper normal limits of ferritin commonly used in the literature [2] and our center. So, this ULN is not extremely high for our men, although it still seems to be unclear whether chosen ULNs is appropriate limit for cellular iron regulation, especially in women. However, there has been no consensus on criteria for hyperfrritinemia till now, as far as we know. Whatever the sufficient answers to reviewer’s question in the case of men, we are very sorry that our responses may seem to be insufficient for chosen ULNs (200 ng/mL) for women, as reviewer pointed out. Therefore, we performed additional analysis of “hyper” ferritin to divide the groups at the 80tprecenties for women, as reviewer’ comments. The ferritin level of 80tprecenties for men and women was 297 ng/mL (nearly consistent with our initial cut off level for hyperferritinmia) and 76 ng/mL, respectively In women, the prevalence of hyperferritinemia was 20.3% (n=9,784) and the aORs for hyperferritinemia across decreasing quartiles of lung function were tended to increase, compared with the highest quartile but did not reach statistical significance.

|  | **Model 1** | | | **Model 2** | | | **Model 3** | | |
| --- | --- | --- | --- | --- | --- | --- | --- | --- | --- |
|  | **OR (95% CI)** | **p value** | **p for trend** | **OR (95% CI)** | **p value** | **p for trend** | **OR (95% CI)** | **p value** | **p for trend** |
| **Hyperferritinemia (n=48,272)** |  |  |  |  |  |  |  |  |  |
| **Quartile of FVC% predicted** |  |  | **<0.001** |  |  | **<0.001** |  |  | **0.009** |
| **Q1 (≤92%, n=11,762, 24.4%)** | **1.149 (1.080-1.222)** | **<0.001** |  | **1.166 (1.096-1.241)** | **<0.001** |  | **1.084 (1.009-1.165)** | **0.028** |  |
| **Q2 (93-98%, n=10,090, 20.9%)** | **1.047 (0.981-1.117)** | **0.170** |  | **1.055 (0.989-1.126)** | **0.105** |  | **1.040 (0.966-1.121)** | **0.298** |  |
| **Q3 (99-106%, n=13,218, 27.4%)** | **0.979 (0.921-1.041)** | **0.496** |  | **0.983 (0.925-1.045)** | **0.590** |  | **0.971 (0.06-1.041)** | **0.410** |  |
| **Q4 (≥107%, n=13,202, 27.3%)** | **1** |  |  | **1** |  |  | **1** |  |  |
| **Quartile of FEV1% predicted** |  |  | **<0.001** |  |  | **<0.001** |  |  | **0.017** |
| **Q1 (≤93%, n=11,355, 23.5%)** | **1.114 (1.047-1.186)** | **0.001** |  | **1.125 (1.057-1.197)** | **<0.001** |  | **1.077 (1.002-1.158)** | **0.044** |  |
| **Q2 (94-98%, n=10,125, 21.0%)** | **1.053 (0.987-1.124)** | **0.115** |  | **1.060 (0.993-1.131)** | **0.078** |  | **1.051 (0.975-1.132)** | **0.194** |  |
| **Q3 (99-106%, n=13,657, 28.3%)** | **0.977 (0.919-1.038)** | **0.447** |  | **0.980 (0.923-1.042)** | **0.521** |  | **0.988 (0.922-1.059)** | **0.743** |  |
| **Q4 (≥107%, n=13,135, 27.2%)** | **1** |  |  | **1** |  |  | **1** |  |  |

Interestingly, sex differences in the association of serum ferritin with various diseases was found in previous studies [6, 12, 43, 44] , as is associated with risk for the disease in men but not in women. This difference seems to be the result of sex differences in serum ferritin levels. The serum ferritin level is generally markedly higher in men than in women, and serum ferritin decreased with age in men but increased with age in women [6]. In line with this, sex difference and greater gaps in serum ferritin level between men and women in our cohort can contribute to much higher lifetime iron store in men than in women, compared to those in previous studies [31-34]. Another reason for this difference might be due to the ef­fects of female sex hormones. The female sex hormones, may contribute to the pathogenesis of disease or serve as protective factors [45, 46]. Taken together, female sex hormones may have a more influence on the health of females than ferritin in the real world. For that reason, we believed that sex segregation analyses are needed in order to yield more meaningful results, especially in middle aged population like our study population, as we already mentioned in discussion. So, we really want you to understand our intension for this study and let us allow to describe the manuscripts only for men, as we submitted initially.

Once again, we really appreciate for your constructive and meaningful comments. We added sentences to explain the our cohort’s characteristics to influence on ferritin levels in the manuscripts as follows;

**(from line 91 on page 4 to line 102 on page 5);**

Of these, we excluded women because the proportion of women with hyperferritinemia, defined as > 200 mg/dL, was only 0.9%, leaving too few women for us to obtain statistically valid results. Thus, we decided to analyze the data from 47,981 men (Fig 1).

🡪

The reference range for serum ferritin is usually from 30 to 300 ng/mL in men and from 15 to 200 ng/mL in women. These ranges seem to vary across reference populations and according to age and sex, which are the most important determinants of serum ferritin [2]. There has been no consensus on criteria for hyperferritinemia. Our cohort consisted of mostly middle aged Asians with higher socioeconomic and education levels and urban habitation with the highest annual PM concentrations in the world. Thus, these characteristics can contribute to more increase in the amount of mobilized iron especially in men than women in our cohort [3-9]. The large difference in serum ferritin level between men and women can contribute to much higher lifetime iron stores in men than in women. Therefore, the prevalence of hyperferrtinemia can be markedly different between men and women. Accordingly, we excluded women due to the small proportion (451, 0.9%) with hyperferritinemia, defined as > 200 ng/mL, leaving too few women for us to obtain statistically valid results. Thus, we decided to analyze the data from 42,927 men (Fig. 1)

C3

The FVC and FEV1 results are transposed between the results stated in the Abstract and those shown in Table 1. Whichever is correct, it would be very unusual in a near normal population to have a 20% difference (93% vs 113%) between the mean %predicted FEV1 and FVC. This would mean that the average FEV1/FVC ratio would have to be either very high or very low, but the reported value of 82 is normal, assuming that is the actual ratio (not %pred). Preferably, it could be reported as 0.82 to eliminate any potential uncertainty for the reader. It seems likely that there is an error in the formula or calculation of the predicted values (although it is recognized that, if the error is consistent, this would not affect the quartile analysis). It would be helpful to add the actual mean FVC and FEV1 values to Table 1, and the authors also might consider comparing these predictions to the North Asia data from the GLI-2012 predictions (Quanjer. Eur Respir J 2012; 40:1324-43).
(Although I cannot access Ms ref 17, the prediction equations reported in the Ms appear to be correct. Calculations for a 40yo, 170cm, 70kg male result in reasonable values for FVC 4.78, FEV1 3.97 for a ratio of 0.83, so for this example an FVC of 100%pred and an FEV1 of 100%pred would give a ratio of .83, but 93%-113%, either way, would be very different).

R3

Thank you for pointing out this important point. The reviewer makes a very important point.

Thanks to reviewer, we early noticed new provisional version of manuscript on the new equations, as we expected. And also, thank you for pointing our typo out and we apologize for our carelessness and the lack of clarity.

Spirometric values should be compared with the normal predictive values obtained from the same population using the same procedures, because different ethnicity and different procedures are known to influence the spirometry results. Spirometry results can only be clinically acceptable if interpreted using appropriate and reliable reference values. Thus, reference equations for subjects' nationalities or ethnicities is pivotal in interpreting spirometry results. Korean Academy of Tuberculosis and Respiratory Disease (KATRD) recommended reference equations for the Korean population, called Choi's reference equation [47].

Unfortunately, the universally used Choi's reference equations in Korea are not validated for normal Korean populations[47]. Choi's reference values for forced vital capacity (FVC) and forced expiratory volume in 1 second (FEV1) were calculated to be much higher than those from other reference equations [48, 49].

So, many physicians have recognized that it is necessary to validate Choi's reference equation for Korean population and develop new reference equations for the current healthy Korean population, because Choi's reference equations was developed 17 years ago and presented larger differences from the observed values, despite reliability. Finally, new reference equations for Korean populations was published officially in Dec 9. 2019 on JKMS, during we revised this manuscript [50]. (We attached provisional PDF files as you can identify)

New reference equations showed the lowest differences between observed values and predicted values, compared to Choi's equations, GLI 2012, and KHNAES IV equations for Korean populations.[47-49]

New reference equations are more suitable and more reliable to predict lung functions for Korean populations.

KATRD suggest new reference equations for Korean populations [50].

Consequently, we used new predicted FEV1 and FVC values obtained from new reference equations to reflect your concern. Nonetheless, similar results were observed in the analysis based on new predicted FEV1 and FVC.

As you recommend, we added the measured values of FEV1 and FVC to Table 1. We modified all values related to lung function in the manuscript. We also modified equations related to predicted spirometric values in method section. And, we also modified table 1, table 2, table 3 and figure 2.

We modified sentences in the manuscripts as follows;

**(from line 138 to line 139 on page 6);**

Predicted FVC = −4.8434 - (0.00008633 × age^2^ [years]) + (0.05292 × height [cm]) + (0.01095 × weight [kg])

Predicted FEV1 = −3.4132 - (0.0002484 × age^2^ [years]) + (0.04578 × height [cm])

🡪

Predicted FVC (L) = -4.38775 - 0.01184 × age + 0.05547 × height

Predicted FEV1(L) = -2.40147 - 0.02134 × age + 0.04103 × height

We changed the transposed FVC and FEV1 results in the Abstract

**(from line 38 to line 40 on page 2);**

Subjects with hyperferritinemia had a lower FEV1% and FVC% than those with normal ferritin levels (112% *vs.* 113% for FVC% and 92% *vs.* 93% for FEV1%, p<0.001 for both).

🡪

Subjects with hyperferritinemia had lower FEV1% and FVC% than those with normal ferritin level (99% *vs.*100% for FEV1%, p = 0.015 and 98% *vs.* 99% for FVC, p = 0.001).

**(Page 11 in table 1);**

Average FEV1/FVC ratio is the actual ratio of the measured values of FEV1 and FVC, as you pointed out. Again we apologize for our mistakes. We modified all FEV1/FVC ratio as the actual ratio to eliminate any potential uncertainty for the readers.

|  | Normal ferritin | Hyperferritinemia | P-value |
| --- | --- | --- | --- |
| FEV1(L)/FVC(L) actual ratio | 0.82±0.05 | 0.82±0.05 | 0.797 |

C4

DISCUSSION. The discussion of the different findings between this study and Ms refs 5 and 6 should note that these population-based studies looked at lung function in relationship to the full range of ferritin values or to mean/median values, whereas this study looks at the likelihood of very high values. While it is reasonable to think that these two approaches might correlate, that is uncertain and should be discussed along with stating the rationale for the choice made for this study. This data could also be analysed in a fashion similar to ref 5 to make a more ‘apples to apples’ comparison

R4

We do appreciate the reviewer’s comment. As reviewer pointed out, our findings are contrary the results from two population based studies [31, 32], which looked at lung function in relationship to the full range of ferritin. The reasons for these contrast results are unclear. However, serum ferritin level accompanied by a different composition of study subjects might explain the disagreement. Demographics including age, sex, different ethnic populations and regions can impact serum ferritin concentrations [2, 3, 5, 41, 51]. Our study is a cohort study, not representative population study, only consisting of Korean middle aged healthy men who mostly live in urban area. In contrast, Ghio’s study and Lee’s study may include various subjects who live in diverse regions including in rural areas, due to the nature of representative population study. Finally, previous reports [31, 32], showing a positive association between and lung function, were undertaken in populations where the serum ferritin level were lower than the current study. Actually, serum ferritin level in our study was about three times higher (median :199 ng/mL, mean:220 ng/mL) than Ghio’s study (mean: 74ng/ml) and Lee’s study (median:62.3 ng/mL).

Brigham’s study [33] and McKeever’s study[34] found no association. Unfortunately, we cannot compare baseline demographics of McKeever’s study [34] because of insufficient data, although mean concentration of ferritin (129.3mg/ml) was still lower than ours. This study was conducted using same data set (NHANES III) to Ghio’s study [31]. The mean serum ferritin level (129.3mg/ml) was higher than Ghio’s study. It was also impossible to assume why the mean ferritin level between two studies was markedly different.

Brigham’s study (mean ferritin level was 36.4 ng/mL which was slightly lower than those in our female subgroup (mean 49.6 ng/mL, median: 38.9 ng/mL)) [33] was conducted in only females 12-49 years who was completely different from our healthy men subjects.

As reviewer suggested, we also analyzed in a fashion similar to Ghio’s study [31]. Instead, we conducted sex segregation analyses as we explained before. (Answer2 to C2)

We adjusted for age, BMI, smoking(pack/years), alcohol intake (gram/day) and variables with a *P* value < .05 in the univariate analyses (men: TB,ALT, total cholesterol TG, LDL cholesterol, fasting glucose,HbA1c,HOMA-IR, insulin and hs-CRP; women: TB,ALT, total cholesterol,TG, LDL cholesterol, fasting glucose,HbA1c,insulin and hs-CRP). Multiple linear regression analysis showed significantly negative association between lung function and serum ferritin level only in men, not women. The results for women is similar to Brigham’s study [33], and also confirms and extends previous findings by demonstrating that female sex hormones may have a more influence on the health of females than ferritin in the real world [45, 46].

Multiple linear regression analysis for serum ferritin level and lung function.

|  | LnFerritin | | |
| --- | --- | --- | --- |
|  | β±SE | P-value | R^2^ (adjusted) |
| Men |  |  |  |
| FVC (%) | -0.002±0.000 | <0.001 | 0.090 |
| FEV1 (%) | -0.001±0.000 | 0.009 | 0.089 |
|  | | | |
| Women |  |  |  |
| FVC (%) | -0.001±0.000 | 0.079 | 0.124 |
| FEV1 (%) | -0.001±0.000 | 0.080 | 0.123 |

Interestingly, previous studies showed a threshold effect of ferritin with incident type 2 diabetes [52, 53]. Like this, the biological effects of ferritin on lung function may also be attributed to a threshold effect in which ferritin levels above a specific threshold are associated with impaired lung function and vice versa. There is biologically plausible explanation for this relationship. While iron is an essential nutrient utilized in almost every aspect of normal cell function, because of its the abilities to participate in redox chemistry, this same feature of iron also makes it dangerous in the generation of hydroxyl radicals and superoxide anions [54]. Ferritin is a specialized iron storage protein that serve as a cellular anti-oxidant and also presents a paradox [22, 54]. If exceed the limit for cellular iron regulation, ferritin released from damaged cell could result in elevated serum ferritin concentration, losing most of its iron and leaving free iron [22]. This serum ferritin’s elevation after disruption of iron homeostasis could be an indirect indicator of iron related oxidative damage to lung [9]. Therefore, the regulation of iron acquisition, utilization, and storage becomes vitally important, perhaps more so in lung than in any other organs, given the double-edged characteristics of ferritin and high local oxygen tensions in the lung. Consequently, there might be watershed to confer respiratory damage from protection, although the accurate threshold level is not determined. We assume that the cohort including more individuals with low serum ferritin level and lower proportion of subjects with ferritin level above the reference range showed the positive association [31, 32] between lung function and ferritin level by enhancing detoxification of iron, and vice versa (as in current study).Considering, the double-edged characteristics of ferritin, we think that evaluation for a threshold effect of ferritin on lung function should be necessary, although dichotomizing ferritin as such may decrease power and obscure the ability to detect subtle differences that are possible when analyzing ferritin as a continuous measure. Of course, there is debate about whether the cut-point of the upper normal limit of ferritin should be appropriate threshold. Unfortunately, there has been no consensus on the definition for hyperfrritinemia, till now. So, we are really want you to let us allow to keep our original analytic methods.

As you suggested, we described the discussion of the different findings between current study and previous studies and rationale for disagreement in discussion section

**(from line 283 on page 23 to line 306 on page 24);**

The mixed results from the previous studies and this study complicate the interpretation of whether ferritin has a beneficial or noxious effect on lung function. Our finding is biologically plausible. Iron must bind to proteins to prevent tissue damage from free radical formation [22, 54]. Consequently, a delicate homeostasis of iron is important. Ferritin is a key protein in iron homeostasis and also presents a paradox. Its capacity to prevent iron’s pro-oxidant activity by oxidizing and sequestering the metal suggest that it might play an important role as an antioxidant and be a marker of oxidative stress and chronic inflammation[21, 22, 54]. Serum ferritin itself is considered benign within the range of homeostasis. However, irrespective of iron store regulation, ferritin can be increased by various inhalants associated with oxidative stress and inflammatory processes[32, 55], and that can result in iron accumulation both locally in the lungs and systemically[21], with consequent cell damage[22]. Serum ferritin leaks from damaged cells, losing most of its iron and leaving free iron[22] that can cause iron-related oxidative damage[22, 54]. Free iron beyond what the body can adequately detoxify could accumulate in the airway epithelium, causing inflammation through superoxide generation and leading to permanent loss of lung function over time [30, 56].Thus, systemic inflammation might be the link between hyperferritinemia and early perturbations in lung function.

🡪

The mixed results from the previous studies [31, 32] and this study complicate a conclusion on whether ferritin may have a beneficial or noxious effect on lung function. The reasons for the mixed results are unclear. However, serum ferritin level in a different composition of study subjects might explain the disagreement. Actually, serum ferritin level in our study was about three times higher than those in previous studies. Interestingly, previous studies showed a threshold effect of ferritin with incident type 2 diabetes [52, 53]. Like this, the biological effects of ferritin on lung function may also be attributed to a threshold effect in which the cohort including more individuals with low serum ferritin level showed a positive association [31, 32] between lung function and ferritin level by enhancing detoxification of iron, and vice versa. There is a biologically plausible explanation for this relationship and our findings. Iron must bind to proteins to prevent tissue damage from free radical formation [22, 54]. Ferritin is a key protein in iron homeostasis and presents a paradox. The capacity of ferritin to prevent iron’s pro-oxidant activity by oxidizing and sequestering the metal suggests that it might play an important role as an antioxidant within the range of homeostasis and be a marker of iron-related oxidative toxicity after disruption of iron homeostasis [9, 22, 54]. Consequently, a delicate homeostasis of iron is vitally important, perhaps more so in lung than in any other organs, given the compounded damage of ferritin and high local oxygen tensions in the lung. If exceeding the limit for cellular iron regulation irrespective of iron store regulation, ferritin released from damaged cells could result in elevated serum ferritin concentration, losing most of its iron and leaving free iron [22]. Free iron beyond what the body can adequately detoxify could accumulate both locally in the airway epithelium and systemically [21], causing subsequent oxidative damage [22, 54], through superoxide generation and leading to permanent loss of lung function over time [30, 56]. Thus, iron-related oxidative damage might be the link between hyperferritinemia and early perturbations in lung function. The biological effects of ferritin on lung function may be attributed to a threshold effect on protection from respiratory damage, although the accurate threshold level is not known.

C5.

CONCLUSION. The introduction proposes a dichotomy of hyper-ferritinemia as a marker of iron stores vs as a marker of systemic inflammation. The absence of an association of iron and iron saturation with lower lung function leads to the conclusion (p24 line 150) that the observed relationship of hyper-ferritinemia to lower lung function is “a result of inflammation”. However, Model 3 adjusted for several other markers of systemic inflammation which might be expected to eliminate the association, but instead only reduced the positive odds ratios modestly (21 and 30% for FEV1 and FVC). The implications of this deserves comment in Discussion.

R5

Thank you for your helpful advice to correct our contradiction. And, we do really appreciate to realize what we said was logically wrong. As reviewer pointed out, adjustment for systemic inflammation marker (especially in hs-CRP) in the final multivariate model did not alter the association of lung function with hyperferritinemia.

Inflammation is thought to play a key role in various chronic diseases and decline of lung function [22, 30] Furthermore, inflammation plays an important role in the regulation of ferritin synthesis and secretion [57]. Interestingly, the independent predictive value of hs-CRP has recently been questioned in cardio-metaboic diseases [26, 52, 58] in line with our findings. This might implicate that ferritin mainly increases the risk of decline of lung function and cardio-metaboic diseases through another various mechanisms besides systemic inflammation.

Generally, elevated levels of ambient particulate matter increase proinflammatory cytokines such as IL-1 , IL-6, TNF-α and GM-CSF [59] IL-6 especially could enter the circulation and stimulate the production of CRP and other systemic inflammation mediators[59]. And, tumor necrosis factor (TNF)-α and IL-6 causes hypoadiponectinemia related to systemic inflammation [60]. However, the bioavailable iron by ambient particulate matter is responsible for reactive oxygen species (oxidative stress) and IL-8 induction (inflammatory mediator) [9].IL-8 is most chemo-attractants for neutrophils and eosinophils and T lymphocytes and contributes to neutrophilic inflammation in chronic bronchitis and emphysema[61], and pathophysiology of allergic airway disease [62]. So, this different pathway for inflammation of IL-6 and IL-8 might be the explanation for no alteration of the association of ferritin and lung function.

Second, the composition of our cohort could be the reason. Our subjects were enrolled from health-screening centers. Thus they were in a higher socioeconomic class and had a more invested interest in their health Despite the fact that hs-CRP is a sensitive marker, it could be influenced by several conditions such as abdominal obesity, high body fat, waist circumference and lipid markers [63]. Therefore, this seems to be the reason for no alteration of the association of ferritin and lung function.

As ferritin is an acute-phase reactant, it reflects not only body iron stores but also subclinical inflammation.

Systemic inflammation could be an important mediator of hyperferritinemia [52, 57], decreased lung function [30],and various chronic diseases[22]. However, the association of lung function with hyperferritinemia remained significant even after adjusting for confounding variables including hsCRP in our study. This might implicate that ferritin increases the risk of decline in lung function through a metabolic oxidative stress, besides systemic inflammation. Therefore, Ferritin is a robust biomarker for lung function in our study population, but no associations of iron and transferrin saturation with lung function. This results lead us to postulate that mechanisms independent of body iron status are involved in the association between ferritin and lung function.

We modified sentences to reflect your concern in the revised manuscripts.

**(from line 324 to line 337 on page 25);**

Ferritin is not only a marker of body iron stores but also an acute-phase reactant that can fluctuate in response to inflammatory mediators and metabolic deficiencies[22]. TSAT is another marker of iron stores and acute-phase reactivity because transferrin can also be elevated in inflammation, which would lower TSAT if circulating iron is constant [64]. However, transferrin levels are less affected by inflammation than ferritin levels [65].Therefore, the discordance between associations of ferritin with cardio-metabolic diseases or lung function and the absence of such an association with other biomarkers of iron metabolism suggests that the elevated ferritin levels seen with cardio-metabolic diseases and decreased lung function could result primarily from inflammatory mechanisms that reflect oxidative stress independent of iron stores.

🡪

Ferritin is not only a marker of body iron stores, but also an acute-phase reactant that can fluctuate in response to inflammatory mediators and metabolic stress [22]. Systemic inflammation could be an important mediator of hyperferritinemia [52, 57] and decreased lung function [30]. However, the association of lung function with hyperferritinemia remained significant in the current study, even after adjusting for several other markers related to systemic inflammation , in line with previous studies[26, 52]. The bioavailable iron is responsible for reactive oxygen species (oxidative stress) and IL-8 induction (inflammatory mediator)[9]. IL-8 is highly chemo-attractive for neutrophils, eosinophils, and T lymphocytes [61, 62], in contrast to IL-6, which is the chief stimulator of CRP production. This might indicate that ferritin increases the risk of decline in lung function through metabolic oxidative stress other than systemic inflammation. Therefore, ferritin is a robust biomarker for lung function in our study population, but no associations were noted for other biomarkers of iron metabolism with lung function. These results lead us to postulate that the elevated ferritin levels seen with cardio-metabolic diseases and decreased lung function could result primarily from metabolic stress that reflects oxidative damage independent of iron stores.

We also modified sentence in the revised manuscripts.

**(from line 27 to line 28 on page 2);**

Elevated ferritin and decreased lung function both predispose people to cardio-metabolic disease, in the light of sharing inflammatory process

🡪

Though elevated ferritin level and decreased lung function both predispose people to cardio-metabolic disease, few reports have investigated the association between them.

**(from line 52 to line 54 on page 3);**

However, ferritin can become elevated in the presence of inflammation irrespective of iron status, and inflammation can contribute to various cardio-metabolic diseases[22].

🡪

Ferritin also can become elevated in the presence of oxidative stress and inflammation irrespective of iron status and can contribute to various clinical diseases, especially pulmonary and cardio-metabolic diseases [22, 24].

**(from line 54 to line 55 on page 3);**

Decreased lung function is also associated with systemic inflammation [30].

🡪

Moreover, decreased lung function is associated with oxidative stress and systemic inflammation [30]

**Minor Comments:**

C6

Table 1 Ferritin level by quartiles for FEV1 shows units of ng/mL while for FVC and elsewhere mg/dL is used. Terminology should be consistent throughout the Ms, tables and figures.

R1

We apologize for our carelessness and the lack of clarity Thank you for pointing our typo out. We changed “mg/dL” into “ng/mL” to unify unit for serum ferritin.

C7

Table 2 It appears that no exclusions were made based upon lung function data. <3% with FEV1/FVC <70 were included. No mention is made of possible early restrictive disease (which has also been associated with higher ferritin levels) in the study population. With a mean FVC of 92+/-10 %pred and a 1st quartile < 86%pred it is likely that some would meet a spirometric criteria for restriction, but as noted in comment 3 above the accuracy of those %pred values is uncertain.

R7

Thank you for pointing out our mistakes. Again, we apologize for our carelessness.

We originally should have excluded the subjects with abnormal ventilation pattern on the spirometric results, because we initially aimed to determine the association between lung function and ferritin only in healthy subjects without overt clinical diseases, abnormal chest X-ray findings and abnormal lung function. We excluded 5,054 subjects with abnormal ventilation pattern. So, we reanalyze the data from 42,927 men. And we also omitted values with FEV1/FVC <70 in manuscript and tables, because there was no subjects with obstructive pattern. Again, we apologize for the lack of clarity. As we responded to C3, we modified spirometric values using new reference equations[50], to reflect your concern.

C8

p21 line 67 The sentence “Although a positive relationship between ferritin and lung function was found…” is accurate, but may not convey its meaning to the reader – could it mean a positive relationship with a decrement in lung function? (I pulled the ref to be sure.) Perhaps the sentence might begin “Contrary to our findings, a positive relationship…” or end explicitly “….two studies, with increased ferritin associated with higher lung function.”

R8

We do appreciate the reviewer’s comment. We agree with reviewer’s opinion. As you suggested, we changed the sentence in the manuscripts as follows;

**(from line 250 to line 252 on page 22);**

Although a positive relationship between ferritin and lung function was found in two studies [31, 32], the others showed that lung function did not correlate with ferritin [33, 34].

🡪

Contrary to our findings, a positive relationship between ferritin and lung function was found in two studies [31, 32], although the other previous studies showed that lung function did not correlate with ferritin [33, 34].

C9

p22 line 95 Since this study is not designed to show causality, it would be more accurate to state “whether ferritin may have a beneficial or noxious effect…”

R9

We do appreciate the reviewer’s comment. We understand reviewer’s oncern.

As you suggested, we changed the sentence in the manuscripts as follows;

**(from line 283 to line 284 on page 23);**

The mixed results from the previous studies and this study complicate the interpretation of whether ferritin has a beneficial or noxious effect on lung function

🡪

The mixed results from the previous studies [31, 32] and this study complicate a conclusion on whether ferritin may have a beneficial or noxious effect on lung function

**References**

1.	. Standardization of Spirometry, 1994 Update. American Thoracic Society. Am J Respir Crit Care Med. 1995; 152: 1107-1136.

2.	Adams PC, Barton JC. A diagnostic approach to hyperferritinemia with a non-elevated transferrin saturation. J Hepatol. 2011; 55: 453-458.

3.	North CM, Rice MB, Ferkol T, Gozal D, Hui C, Jung SH *et al.* Air pollution in the Asia-Pacific Region: A Joint Asian Pacific Society of Respirology/American Thoracic Society perspective (Republication). Respirology. 2019; 24: 484-491.

4.	Park JH, Hong IY, Chung JW, Choi HS. Vitamin D status in South Korean population: Seven-year trend from the KNHANES. Medicine (Baltimore). 2018; 97: e11032.

5.	Seong JM, Yoon YS, Lee KS, Bae NY, Gi MY, Yoon H. Gender difference in relationship between serum ferritin and 25-hydroxyvitamin D in Korean adults. PLoS One. 2017; 12: e0177722.

6.	Gye HJ, Kim JM, Yoo C, Shim SH, Won YS, Sung KC *et al.* Relationship between high serum ferritin level and glaucoma in a South Korean population: the Kangbuk Samsung health study. Br J Ophthalmol. 2016; 100: 1703-1707.

7.	Choi HS, Oh HJ, Choi H, Choi WH, Kim JG, Kim KM *et al.* Vitamin D insufficiency in Korea--a greater threat to younger generation: the Korea National Health and Nutrition Examination Survey (KNHANES) 2008. J Clin Endocrinol Metab. 2011; 96: 643-651.

8.	Holick MF. Vitamin D deficiency. N Engl J Med. 2007; 357: 266-281.

9.	Ball BR, Smith KR, Veranth JM, Aust AE. Bioavailability of iron from coal fly ash: mechanisms of mobilization and of biological effects. Inhal Toxicol. 2000; 12 Suppl 4: 209-225.

10.	Malavolti M, Mussi C, Poli M, Fantuzzi AL, Salvioli G, Battistini N *et al.* Cross-calibration of eight-polar bioelectrical impedance analysis versus dual-energy X-ray absorptiometry for the assessment of total and appendicular body composition in healthy subjects aged 21-82 years. Ann Hum Biol. 2003; 30: 380-391.

11.	Wen CP, David Cheng TY, Tsai SP, Chan HT, Hsu HL, Hsu CC *et al.* Are Asians at greater mortality risks for being overweight than Caucasians? Redefining obesity for Asians. Public Health Nutr. 2009; 12: 497-506.

12.	Sung KC, Kang SM, Cho EJ, Park JB, Wild SH, Byrne CD. Ferritin is independently associated with the presence of coronary artery calcium in 12,033 men. Arterioscler Thromb Vasc Biol. 2012; 32: 2525-2530.

13.	Park SK, Choi WJ, Oh CM, Kim J, Shin H, Ryoo JH. Association between serum ferritin levels and the incidence of obesity in Korean men: a prospective cohort study. Endocr J. 2014; 61: 215-224.

14.	Matthews DR, Hosker JP, Rudenski AS, Naylor BA, Treacher DF, Turner RC. Homeostasis model assessment: insulin resistance and beta-cell function from fasting plasma glucose and insulin concentrations in man. Diabetologia. 1985; 28: 412-419.

15.	Morrison HI, Semenciw RM, Mao Y, Wigle DT. Serum iron and risk of fatal acute myocardial infarction. Epidemiology. 1994; 5: 243-246.

16.	Song JU, Jang Y, Lim SY, Ryu S, Song WJ, Byrne CD *et al.* Decreased lung function is associated with risk of developing non-alcoholic fatty liver disease: A longitudinal cohort study. PLoS One. 2019; 14: e0208736.

17.	Song JU, Hwang J, Ahn JK. Serum uric acid is positively associated with pulmonary function in Korean health screening examinees. Mod Rheumatol. 2017; 27: 1057-1065.

18.	Kuang Y, Wang Q. Iron and lung cancer. Cancer Lett. 2019; 464: 56-61.

19.	Alvarez SW, Sviderskiy VO, Terzi EM, Papagiannakopoulos T, Moreira AL, Adams S *et al.* NFS1 undergoes positive selection in lung tumours and protects cells from ferroptosis. Nature. 2017; 551: 639-643.

20.	Vaupel P, Kallinowski F, Okunieff P. Blood flow, oxygen and nutrient supply, and metabolic microenvironment of human tumors: a review. Cancer Res. 1989; 49: 6449-6465.

21.	Ghio AJ, Hilborn ED, Stonehuerner JG, Dailey LA, Carter JD, Richards JH *et al.* Particulate matter in cigarette smoke alters iron homeostasis to produce a biological effect. Am J Respir Crit Care Med. 2008; 178: 1130-1138.

22.	Kell DB, Pretorius E. Serum ferritin is an important inflammatory disease marker, as it is mainly a leakage product from damaged cells. Metallomics. 2014; 6: 748-773.

23.	Holgate ST. Air pollution: The time has arrived for the medical profession to take ownership of the problem and act. Respirology. 2019; 24: 1138-1139.

24.	Zhang WZ, Butler JJ, Cloonan SM. Smoking-induced iron dysregulation in the lung. Free Radic Biol Med. 2019; 133: 238-247.

25.	Podmore C, Meidtner K, Schulze MB, Scott RA, Ramond A, Butterworth AS *et al.* Association of Multiple Biomarkers of Iron Metabolism and Type 2 Diabetes: The EPIC-InterAct Study. Diabetes Care. 2016; 39: 572-581.

26.	Yeap BB, Divitini ML, Gunton JE, Olynyk JK, Beilby JP, McQuillan B *et al.* Higher ferritin levels, but not serum iron or transferrin saturation, are associated with Type 2 diabetes mellitus in adult men and women free of genetic haemochromatosis. Clin Endocrinol (Oxf). 2015; 82: 525-532.

27.	Park SK, Ryoo JH, Kim MG, Shin JY. Association of serum ferritin and the development of metabolic syndrome in middle-aged Korean men: a 5-year follow-up study. Diabetes Care. 2012; 35: 2521-2526.

28.	Salonen JT, Nyyssonen K, Korpela H, Tuomilehto J, Seppanen R, Salonen R. High stored iron levels are associated with excess risk of myocardial infarction in eastern Finnish men. Circulation. 1992; 86: 803-811.

29.	Engstrom G, Hedblad B, Nilsson P, Wollmer P, Berglund G, Janzon L. Lung function, insulin resistance and incidence of cardiovascular disease: a longitudinal cohort study. J Intern Med. 2003; 253: 574-581.

30.	Shaaban R, Kony S, Driss F, Leynaert B, Soussan D, Pin I *et al.* Change in C-reactive protein levels and FEV1 decline: a longitudinal population-based study. Respir Med. 2006; 100: 2112-2120.

31.	Ghio AJ, Hilborn ED. Indices of iron homeostasis correlate with airway obstruction in an NHANES III cohort. Int J Chron Obstruct Pulmon Dis. 2017; 12: 2075-2084.

32.	Lee CH, Goag EK, Lee SH, Chung KS, Jung JY, Park MS *et al.* Association of serum ferritin levels with smoking and lung function in the Korean adult population: analysis of the fourth and fifth Korean National Health and Nutrition Examination Survey. Int J Chron Obstruct Pulmon Dis. 2016; 11: 3001-3006.

33.	Brigham EP, McCormack MC, Takemoto CM, Matsui EC. Iron status is associated with asthma and lung function in US women. PLoS One. 2015; 10: e0117545.

34.	McKeever TM, Lewis SA, Smit HA, Burney P, Cassano PA, Britton J. A multivariate analysis of serum nutrient levels and lung function. Respir Res. 2008; 9: 67.

35.	. Chronic Obstructive Pulmonary Disease: A Disorder of the Cardiovascular and Respiratory Systems. Lund, Sweden, April 15-16, 2004. Proceedings. Proc Am Thorac Soc. 2005; 2: 5-100.

36.	Hole DJ, Watt GC, Davey-Smith G, Hart CL, Gillis CR, Hawthorne VM. Impaired lung function and mortality risk in men and women: findings from the Renfrew and Paisley prospective population study. BMJ. 1996; 313: 711-715; discussion 715-716.

37.	Liu Z, Ye F, Zhang H, Gao Y, Tan A, Zhang S *et al.* The association between the levels of serum ferritin and sex hormones in a large scale of Chinese male population. PLoS One. 2013; 8: e75908.

38.	Hou Y, Zhang S, Wang L, Li J, Qu G, He J *et al.* Estrogen regulates iron homeostasis through governing hepatic hepcidin expression via an estrogen response element. Gene. 2012; 511: 398-403.

39.	Wehr E, Pilz S, Boehm BO, Marz W, Obermayer-Pietsch B. Association of vitamin D status with serum androgen levels in men. Clin Endocrinol (Oxf). 2010; 73: 243-248.

40.	Parikh G, Varadinova M, Suwandhi P, Araki T, Rosenwaks Z, Poretsky L *et al.* Vitamin D regulates steroidogenesis and insulin-like growth factor binding protein-1 (IGFBP-1) production in human ovarian cells. Horm Metab Res. 2010; 42: 754-757.

41.	Adams PC, Reboussin DM, Barton JC, McLaren CE, Eckfeldt JH, McLaren GD *et al.* Hemochromatosis and iron-overload screening in a racially diverse population. N Engl J Med. 2005; 352: 1769-1778.

42.	Ma Y, Kawabata T, Hamazaki S, Ogino T, Okada S. Sex differences in oxidative damage in ddY mouse kidney treated with a renal carcinogen, iron nitrilotriacetate. Carcinogenesis. 1998; 19: 1983-1988.

43.	Piperno A, Trombini P, Gelosa M, Mauri V, Pecci V, Vergani A *et al.* Increased serum ferritin is common in men with essential hypertension. J Hypertens. 2002; 20: 1513-1518.

44.	von Hanno T, Bertelsen G, Broderstad AR, Wilsgaard T, Mathiesen EB. Serum ferritin and hemoglobin are independently associated with wider retinal venular caliber: the Tromso Study 2001-2008. Invest Ophthalmol Vis Sci. 2013; 54: 7053-7060.

45.	Clegg D, Hevener AL, Moreau KL, Morselli E, Criollo A, Van Pelt RE *et al.* Sex Hormones and Cardiometabolic Health: Role of Estrogen and Estrogen Receptors. Endocrinology. 2017; 158: 1095-1105.

46.	Caracta CF. Gender differences in pulmonary disease. Mt Sinai J Med. 2003; 70: 215-224.

47.	Choi JK, Paek D, Lee JO. Normal predictive values of spirometry in Korean population. Tuberculosis and Respiratory Diseases. 2005; 58: 230-242.

48.	Quanjer PH, Stanojevic S, Cole TJ, Baur X, Hall GL, Culver BH *et al.* Multi-ethnic reference values for spirometry for the 3-95-yr age range: the global lung function 2012 equations. Eur Respir J. 2012; 40: 1324-1343.

49.	Eom SY, Kim H. Reference values for the pulmonary function of Korean adults using the data of Korea National Health and Nutrition Examination Survey IV (2007-2009). J Korean Med Sci. 2013; 28: 424-430.

50.	Choi HS, Park YB, Yoon HK, Lim SY, Kim T-H, Park JH *et al.* Validation of Previous Spirometric Reference Equations and New Equations. J Korean Med Sci. 2019; 34.

51.	Yu HJ, Kwon MJ, Woo HY, Park H. Analysis of 25-Hydroxyvitamin D Status According to Age, Gender, and Seasonal Variation. J Clin Lab Anal. 2016; 30: 905-911.

52.	Jung CH, Lee MJ, Hwang JY, Jang JE, Leem J, Park JY *et al.* Elevated serum ferritin level is associated with the incident type 2 diabetes in healthy Korean men: a 4 year longitudinal study. PLoS One. 2013; 8: e75250.

53.	Forouhi NG, Harding AH, Allison M, Sandhu MS, Welch A, Luben R *et al.* Elevated serum ferritin levels predict new-onset type 2 diabetes: results from the EPIC-Norfolk prospective study. Diabetologia. 2007; 50: 949-956.

54.	Arosio P, Levi S. Ferritin, iron homeostasis, and oxidative damage. Free Radic Biol Med. 2002; 33: 457-463.

55.	Koorts AM, Viljoen M. Ferritin and ferritin isoforms II: protection against uncontrolled cellular proliferation, oxidative damage and inflammatory processes. Arch Physiol Biochem. 2007; 113: 55-64.

56.	Pittet JF, Mackersie RC, Martin TR, Matthay MA. Biological markers of acute lung injury: prognostic and pathogenetic significance. Am J Respir Crit Care Med. 1997; 155: 1187-1205.

57.	Torti FM, Torti SV. Regulation of ferritin genes and protein. Blood. 2002; 99: 3505-3516.

58.	Danesh J, Wheeler JG, Hirschfield GM, Eda S, Eiriksdottir G, Rumley A *et al.* C-reactive protein and other circulating markers of inflammation in the prediction of coronary heart disease. N Engl J Med. 2004; 350: 1387-1397.

59.	van Eeden SF, Tan WC, Suwa T, Mukae H, Terashima T, Fujii T *et al.* Cytokines involved in the systemic inflammatory response induced by exposure to particulate matter air pollutants (PM(10)). Am J Respir Crit Care Med. 2001; 164: 826-830.

60.	Fantuzzi G. Adipose tissue, adipokines, and inflammation. J Allergy Clin Immunol. 2005; 115: 911-919; quiz 920.

61.	Keatings VM, Collins PD, Scott DM, Barnes PJ. Differences in interleukin-8 and tumor necrosis factor-alpha in induced sputum from patients with chronic obstructive pulmonary disease or asthma. Am J Respir Crit Care Med. 1996; 153: 530-534.

62.	Erger RA, Casale TB. Interleukin-8 is a potent mediator of eosinophil chemotaxis through endothelium and epithelium. Am J Physiol. 1995; 268: L117-122.

63.	Zeba AN, Delisle HF, Rossier C, Renier G. Association of high-sensitivity C-reactive protein with cardiometabolic risk factors and micronutrient deficiencies in adults of Ouagadougou, Burkina Faso. Br J Nutr. 2013; 109: 1266-1275.

64.	Wish JB. Assessing iron status: beyond serum ferritin and transferrin saturation. Clin J Am Soc Nephrol. 2006; 1 Suppl 1: S4-8.

65.	Szoke D, Panteghini M. Diagnostic value of transferrin. Clin Chim Acta. 2012; 413: 1184-1189.
