# Supplementary material for: Decreased lung function is associated with elevated ferritin but not iron or transferrin saturation in 42,927 healthy Korean men: A cross-sectional study
Source: PLoS One. 2020 Apr 2;15(4):e0231057. doi: 10.1371/journal.pone.0231057 (PMC7117746; doi:10.1371/journal.pone.0231057)
Supplement: Attachment — Submitted filename: Response to Reviewers.docx [file pone.0231057.s002.docx]

**Manuscript ID : PONE-D-19-26396**

**Title: Decreased lung function is associated with elevated ferritin, but not iron or transferrin saturation in 42,927 healthy Korean men: a cross-sectional study**

On behalf of my co-authors, I thank you for the constructive comments and criticisms of our manuscript named above. In response to the reviewer’s suggestions and questions, we have revised the manuscript as suggested. We have responded to the reviewer’s comments in a point-by-point manner below. Please find enclosed the revised version of the manuscript. We hope we have dealt with comments of the reviewers to their satisfaction.

We hope that our revised manuscript is now suitable for publication and look forward to your response.

PS)

Lastly, we wish you to allow to modify financial support as bellows;

We apologize for our mistakes and carelessness.

**Financial Support:** The 2019 scientific promotion program funded by Jeju National University.

🡪

**Financial Support:** The 2020 scientific promotion program funded by Jeju National University.

With best wishes

Jae-Uk Song, MD, PhD,

Division of Pulmonary and Critical Care Medicine, Department of Internal Medicine, Kangbuk Samsung Hospital, Sungkyunkwan University School of Medicine,

29 Saemunan-ro, Jongno-gu, Seoul 03181, Republic of Korea

Phone: +82-2-2001-1597

Fax: +82-2-2001-1596

E-mail: [khfever76@gmail.com](mailto:khfever76@gmail.com)

**Point-by-point responses to reviewer’s comments**

**TO THE COMMENTS OF THE REVIEWER 2.**

The revised study has been modified by the exclusion of subjects with low measured lung function, just over 10% of the prior study group. A new source of reference equations has been chosen resulting in %predicted values which are much more reasonable than the prior report.

**Specific comments:**

C1. Study Design

The criteria used for PFT exclusion should be stated (eg, FVC <80%, FEV1<80%, FEV1/FVC< 0.70).
The last sentence re FEV1/FVC can be deleted here and stated earlier (prior comment).

R1.

We do appreciate the reviewer’s comment. We apologize for our carelessness and the lack of clarity.

As reviewer pointed out, we stated the criteria used for PFT exclusion in the manuscript as follows;

**(from line 85 to line 86 on page 4 );**

We excluded 85,455 participants who had a ventilation disorder on the basis of spirometric results [1]

🡪

We excluded 85,455 participants who had an impaired lung function (a ventilation disorder) (the subjects without normal lung function defined as forced expiratory volume in one second (FEV1) to forced vital capacity (FVC) [FEV1/FVC] ≥ 0.7 and FVC ≥80% predicted) [2]

C2. Lung Fxn measurement

Thank you for changing the Reference data source (although I think there was also a calculation error in the prior version); the predicted values now look much more reasonable. The source is cited (and available online) so it is not necessary to include the equations in the Ms. The last sentence re FEV1/FVC can be deleted here and stated earlier (prior comment).

R2.

Thank you for your advice to improved our manuscript much more. As you recommended, we removed the reference equations in the manuscript. (from line 138 to line 139 on page 6)

And, we apologize for our carelessness. Thank you for pointing our mistakes.

We omitted last sentence in the lung function measurement section. (from line 143 on page 6)

C3. Results

C3-1.

With the large number of subjects it is possible to show statistical significance for very small differences. Whether or not these are clinically or physiologically important is another matter that should be addressed. This would be most appropriate in Discussion, but the presentation of results can also be tempered.

eg, “subjects in the hyperferritinemia group were more likely to be younger”, yet a mean age of 38.7 v 38.1, with wide overlap of SDs, is hardly compelling. Similarly, for smoking (~62% v 60%) and alcohol use (91.2 v 87.4%). The message might be how similar these exposures are despite the wide difference in ferritin levels. These results would be appropriately preceded by “Small but significant differences were seen….” or similar acknowledgement of the small magnitude.

More importantly, the differences in the primary comparison of lung function are also very small in both absolute terms (FVC 4.75 v 4.73; FEV1 3.89 v 3.88) and when adjusted for age and height by % predicted. The latter should be reported to at least one additional place so that rounding does not influence the apparent difference.

R3-1.

Thank you for your reasonable comments. The reviewer makes a very important point. We fully understand reviewer’s concern. We also agree that it could not be clinically meaningful, although it show statistical significance in a large study population. So, we need to tone the presentation of results down, as reviewer pointed out. We modified result section to reflect reviewer’s concern.

**(from line 168 to line 172 on page 7 );**

Compared with the normal ferritin group, subjects in the hyperferritinemia group were more likely to be younger, to have smoked, to drink alcohol, and have higher blood pressure. Serum ferritin level was negatively associated with a quartile increase in FVC% (p=0.001) and FEV1% (p<0.001), but the difference in FEV1 (L)/FVC (L) between the groups was not statistically significant (p=0.797).

🡪

When compared clinical variables between two groups, small but significant differences were seen in age, smoking habit, alcohol intake, liver function, CRP, blood pressure and a variety of metabolic parameters, including BMI, fasting glucose, and HbA1c. Compared with the normal ferritin group, subjects in the hyperferritinemia group were to have lower value of spirometry with a narrow margin, although those were statistically significant. However, the difference in FEV1 (L)/FVC (L) between the groups was not statistically significant (p=0.797).

**(from line 176 on page 7 to line 180 on page 8 );**

A comparison of clinical characteristics between subjects with and without high iron or TSAT is shown in Table 3. Subjects with high iron and TSAT were more likely than others to drink and smoke. However, both high iron and TSAT were inversely associated with hsCRP and metabolic values, including BMI, HbA1c, insulin, and HOMA-IR, although insulin was only significantly related to TSAT

🡪

A comparison of clinical characteristics between subjects with and without high iron or TSAT is shown in Table 3. Subjects with high iron and TSAT were more likely than others to drink and smoke with a slight difference. However, both high iron and TSAT were inversely associated with hsCRP and metabolic values, including BMI, HbA1c, insulin, and HOMA-IR, although insulin was only related to TSAT.

We also described reviewer’s concern in discussion section of the revised manuscripts as below;

**(from line 340 to line 342 on page 26);**

This gave us sufficient statistical power and could be more relevant to healthy population with normal lung functions.

🡪

This gave us sufficient statistical power and could be more relevant to healthy population with normal lung functions. However, it should be considered that it is possible to show statistical significance for small differences in the large sample sized studies. And, our study demonstrated modest association between hyperferritinemia and lung function parameters. There also have been discordant results on this issue among studies [3-6], although those were undertaken in populations where the serum ferritin level were lower than the current study. It is suggested that a variety of factors are likely to have more influence on lung function than ferritin in the real world, and vice versa. Therefore, we cannot exclude the possibility of some unmeasured or residual confounding factors in the association between ferritin and lung function.

C3-2.

It seems mathematically odd here that the FVC and FEV1 values shown for the combined groups match that of the smaller hyper- group rather than that of the normal group making up over 80% of the study population.

R3-2.

We believe the odd FVC and FEV1 values in table 1 might be the rounding off the numbers to three decimal places, as you can see below table. Therefore, we reported the value of FVC and FEV1 to three decimal places to decrease the mathematical add.

|  | **All subjects (n=42,927)** | **Normal ferritin**  **(ferritin ≤300 ng/mL) (n=34,743, 80.7%)** | **Hyperferritinemia**  **(ferritin >300 ng/mL) (n=8,184, 19.3%)** |
| --- | --- | --- | --- |
| **FVC (L) ^†^** | 4.73±0.56  4.734±0.556 | 4.75±0.55  4.745±0.545 | 4.73±0.56  4.730±0.557 |
| **FEV1(L) ^†^** | 3.88±0.47  3.884±0.474 | 3.89±0.47  3.891±0.467 | 3.88±0.48  3.879±0.477 |
| **FVC% predicted^†^** | 99 ± 9  98.62±8.83 | 99 ± 9  98.87±8.88 | 98 ± 9  98.43±8.63 |
| **FEV1% predicted^†^** | 100 ± 9  99.54±9.32 | 100 ± 9  99.61±9.36 | 99 ± 9  99.22±9.12 |

C3-3.

Rounding is also an issue for bilirubin and creatinine, where the reported numbers for the two groups appear identical, but for bilirubin the star indicates a highly significant difference.

R3-3.

And we apologize for our typo. Originally, the value of total bilirubin in hyperferritinemia group was 1.0 ± 0.4 in table 1. We changed the value of total bilirubin in table 1 as bellow;

|  | **All subjects (n=42,927)** | **Normal ferritin**  **(ferritin ≤300 ng/mL) (n=34,743, 80.7%)** | **Hyperferritinemia**  **(ferritin >300 ng/mL) (n=8,184, 19.3%)** | **p value** |
| --- | --- | --- | --- | --- |
| **Total bilirubin (mg/dL) (n=47,980)^*^** | 0.9 ± 0.4 | 0.9 ± 0.4 | 0.9 ± 0.4 | <0.001 |
| **Total bilirubin (mg/dL) (n=47,980)^*^** | 0.9 ± 0.4 | 0.9 ± 0.4 | 1.0 ± 0.4 | <0.001 |

C4.

The lower portion of Table 1 shows a slight inverse relationship of the ferritin levels vs FVC and FEV1 quartiles. This is seen in both the normal and hyper ferritin groups, contrary to the idea that there may be a threshold effect for ferritin to affect lung function. It is not clear what the significance test (“compared with hyperferritinemia”) means here; is there a difference in the relationship between the two?...and if so, which is stronger? For both FVC and FEV1, the % difference in ferritin level from quartile 1 to 4 is greater for the normal group, although the absolute differences are larger in the hyper- group

R4.

We apologize for the lack of clarity. We believe that wrong presentation for significance test could be a result of confusion. Serum ferritin level was decreased across increasing quartile of FVC% (p=0.001) and FEV1% (p<0.001) in all subjects, using Kruskal-Wallis tests. So, marks such as “†” and “*” did not mean significant difference between the two groups (normal ferritin vs. hyperferritinemia). Therefore, we had better remove the lower portion of Table 1 to avoid the confusion. We really appreciate that we had a chance to revise this. Interestingly, inverse relationship of the ferritin levels across increasing quartile of FVC% and FEV1% showed statistical significance in both two groups. Therefore, it seems to be contrary to the idea that there may be a threshold effect for ferritin to affect lung function, as you pointed out. We can’s fully explain the reasons for this. However, the median and mean level of ferritin in our subjects with normal ferritin level was 177ng/ml which was still much higher than Ghio’s study (mean: 74ng/ml) [3] and Lee’s study (median: 62.3 ng/mL) [4] showing a contrary effect for ferritin on the lung function to ours. Thus, the association between ferritin (not dichotomizing ferritin) and lung function might be depend on serum ferritin level of study subjects. Consequently, we still believed threshold effect or double-edged characteristics for ferritin on lung function which could be hypothesis that might explain mixed findings and different serum ferritin level among studies. However, unfortunately the accurate threshold level is not known and there has been no consensus on criteria for hyperfrritinemia till now. Therefore, there could be debate about whether our definition of hyperferritinemia may be appropriate threshold for protection from respiratory damage. We also do not thick that our definition of hyperferritinemia may be appropriate threshold value. We just selected this level, based on the usual upper normal limits of ferritin commonly used in the literature [7] and our center. Besides, careful consideration is required when interpretating the findings from the large sample sized studies, because it is possible to show statistical significance for small differences, as you pointed earlier. Slight inverse relationship of the ferritin levels vs FVC and FEV1 quartiles in the normal ferritin group could not be clinically meaningful, although it show statistical significance. Therefore, further prospective studies are needed to determine whether ferritin is an independent risk of lung function deterioration and what the threshold level is appropriate.

We remove the lower portion of Table 1 to avoid the confusion. Instead, we add some sentences on the our definition of hyperferritinemia and the uncertainty for appropriate threshold level, to reflect your concerns

**(from line 342 on page 26);**

This gave us sufficient statistical power and could be more relevant to healthy population with normal lung functions. However, it should be considered that it is possible to show statistical significance for small differences in the large sample sized studies. And, our study demonstrated modest association between hyperferritinemia and lung function parameters. There also have been discordant results on this issue among studies [3-6], although those were undertaken in populations where the serum ferritin level were lower than the current study. It is suggested that a variety of factors are likely to have more influence on lung function than ferritin in the real world, and vice versa. Therefore, we cannot exclude the possibility of some unmeasured or residual confounding factors in the association between ferritin and lung function. (see response C3-1 which we commented above) Nonetheless, several limitations need to be addressed.

🡪

This gave us sufficient statistical power and could be more relevant to healthy population with normal lung functions. However, it should be considered that it is possible to show statistical significance for small differences in the large sample sized studies. And, our study demonstrated modest association between hyperferritinemia and lung function parameters. There also have been discordant results on this issue among studies [3-6], although those were undertaken in populations where the serum ferritin level were lower than the current study. It is suggested that a variety of factors are likely to have more influence on lung function than ferritin in the real world, and vice versa. Therefore, we cannot exclude the possibility of some unmeasured or residual confounding factors in the association between ferritin and lung function. Furthermore, there has been no consensus on criteria for hyperfrritinemia and, there could be debate about whether our definition of hyperferritinemia may be appropriate threshold for protection from respiratory damage. Even if our study with the large sample size demonstrated significant association between hyperferritinemia and lung function parameters, further prospective studies are needed to determine whether ferritin is an independent risk of lung function deterioration and what the threshold level is appropriate. Our study also had several limitations.

We removed below sentence from line 306 on page 24 to avoid overlapping.

“although the accurate threshold level is not known.”

.

C5. Table 2
The % numbers in title are confusing and should be removed. They show the distribution between groups, while the similarly displayed % values in the table do not, but are based upon the total in each group.

R5.

As you recommended, we removed the % numbers in title. And, we also removed the % numbers at title in table 3. Thank you for your advice.

And we apologize for our typo in table 3. The number of subjects with iron value was 35,890 not 34,873

**References**

**1.**	(1995) Standardization of Spirometry, 1994 Update. American Thoracic Society. Am J Respir Crit Care Med 152: 1107-1136.

**2.**	West JB (2013) GOLD Executive Summary. Am J Respir Crit Care Med 188: 1366-1367.

**3.**	Ghio AJ, Hilborn ED (2017) Indices of iron homeostasis correlate with airway obstruction in an NHANES III cohort. Int J Chron Obstruct Pulmon Dis 12: 2075-2084.

**4.**	Lee CH, Goag EK, Lee SH, Chung KS, Jung JY, Park MS, et al. (2016) Association of serum ferritin levels with smoking and lung function in the Korean adult population: analysis of the fourth and fifth Korean National Health and Nutrition Examination Survey. Int J Chron Obstruct Pulmon Dis 11: 3001-3006.

**5.**	Brigham EP, McCormack MC, Takemoto CM, Matsui EC (2015) Iron status is associated with asthma and lung function in US women. PLoS One 10: e0117545.

**6.**	McKeever TM, Lewis SA, Smit HA, Burney P, Cassano PA, Britton J (2008) A multivariate analysis of serum nutrient levels and lung function. Respir Res 9: 67.

**7.**	Adams PC, Barton JC (2011) A diagnostic approach to hyperferritinemia with a non-elevated transferrin saturation. J Hepatol 55: 453-458.
