# Supplementary material for: Decreased lung function is associated with elevated ferritin but not iron or transferrin saturation in 42,927 healthy Korean men: A cross-sectional study
Source: PLoS One. 2020 Apr 2;15(4):e0231057. doi: 10.1371/journal.pone.0231057 (PMC7117746; doi:10.1371/journal.pone.0231057)
Supplement: Attachment — Submitted filename: Response to Reviewers.docx [file pone.0231057.s003.docx]

**Manuscript ID : PONE-D-19-26396**

**Title: Decreased lung function is associated with elevated ferritin, but not iron or transferrin saturation in 42,927 healthy Korean men: a cross-sectional study**

On behalf of my co-authors, I thank you for the constructive comments and criticisms of our manuscript named above. In response to the reviewer’s suggestions and questions, we have revised the manuscript as suggested. We have responded to the reviewer’s comments in a point-by-point manner below. Please find enclosed the revised version of the manuscript. We hope we have dealt with comments of the reviewers to their satisfaction.

We hope that our revised manuscript is now suitable for publication and look forward to your response.

With best wishes

Jae-Uk Song, MD, PhD,

Division of Pulmonary and Critical Care Medicine, Department of Internal Medicine, Kangbuk Samsung Hospital, Sungkyunkwan University School of Medicine,

29 Saemunan-ro, Jongno-gu, Seoul 03181, Republic of Korea

Phone: +82-2-2001-1597

Fax: +82-2-2001-1596

E-mail: [khfever76@gmail.com](mailto:khfever76@gmail.com)

**Point-by-point responses to reviewer’s comments**

**TO THE COMMENTS OF THE REVIEWER 2.**

C1. Abstract
The FVC and FEV1 values are now more accurately stated in Table 1, but no change was made in the Abstract so that the rounded numbers indicate differences of 1% which is more than double the actual values of ~ 0.4%. These should be restated with the same values as in the table.

R1.

We do appreciate the reviewer’s comment. We apologize for our carelessness.

As reviewer pointed out, we restated with the values of predicted FVC and FEV1 to three decimal places as in the table 1, to avoid the confusion.

**(from line 39 to line 40 on page 2 );**

Subjects with hyperferritinemia had lower FEV1% and FVC% than those with normal ferritin level (99% *vs.*100% for FEV1%, p = 0.015 and 98% *vs.* 99% for FVC, p = 0.001).

🡪

Subjects with hyperferritinemia had lower FEV1% and FVC% than those with normal ferritin level with a slight difference, but those were statistically significant (99.22% *vs.*99.61% for FEV1%, p = 0.015 and 98.43% *vs.* 98.87% for FVC, p = 0.001).

C2. Study design

In describing the excluded subjects it would be more straightforward to state the exclusion (rather than inclusion) criteria, ie
…participants who had lung function impairment, defined as forced expiratory volume in one second (FEV1) to forced vital capacity (FVC) [FEV1/FVC] < 0.7 and FVC < 80% predicted.

R2-A

We do appreciate the reviewer’s comment.

As reviewer pointed out, we stated the exclusion criteria rather than inclusion criteria in the manuscript as follows;

**(from line 86 to line 89 on page 4 );**

We excluded 85,455 participants who had an impaired lung function (the subjects without normal lung function defined as forced expiratory volume in one second (FEV1) to forced vital capacity (FVC) [FEV1/FVC] ≥ 0.7 and FVC ≥80% predicted) [1]

🡪

We excluded 85,455 participants who had a ventilation disorder (pure restriction: forced expiratory volume in one second (FEV1) to forced vital capacity (FVC) [FEV1/FVC] ≥ 0.7 and FVC < 80% predicted; pure obstruction: FEV1/FVC < 0.7 and FVC ≥80% predicted and mixed ventilation disorder: FEV1/FVC < 0.7 and FVC < 80% predicted) [1]

C3. Lung Function measurement
The deletion of the equations, requires proofreading for appropriate changes in the accompanying text, ie
delete “the following” and change : to

R3.

We apologize for our carelessness. Thank you for pointing our mistakes.

As you recommended, we removed “the following” and modified as follows;

And we modified reference because reference article was published officially.

([J Korean Med Sci.](https://www.ncbi.nlm.nih.gov/pubmed/?term=Validation+of+Previous+Spirometric+Reference+Equations+and+New+Equations.) 2019 Dec 9; 34(47):e304)

**(from line 139 to line 141 on page 6 );**

The predicted values for FEV1 and FVC were calculated from the following equations obtained in a representative Korean population sample [2];

🡪

The predicted values for FEV1 and FVC were calculated from equations to obtain in a representative Korean population sample [2].

C4. Results
3rd and 4th sentences are awkwardly stated. Suggest:
Comparison of clinical variables between the two groups showed small, but significant, differences in age, …..
Compared with the normal ferritin group, subjects in the hyperferritinemia group had lower values of spirometry….

R4.

Thank you for your advice. We apologize for our awkward presentation.

As you recommended, we modified sentences as follows;

**(from line 169 to line 173 on page 7 );**

When compared clinical variables between two groups, small but significant differences were seen in age, smoking habit, alcohol intake, liver function, CRP, blood pressure and a variety of metabolic parameters, including BMI, fasting glucose, and HbA1c. Compared with the normal ferritin group, subjects in the hyperferritinemia group were to have lower value of spirometry with a narrow margin, although those were statistically significant.

🡪

Comparison of clinical variables between the two groups showed small, but significant difference in age, smoking habit, alcohol intake, liver function, CRP, blood pressure and a variety of metabolic parameters, including BMI, fasting glucose, and HbA1c. Compared with the normal ferritin group, subjects in the hyperferritinemia group had lower values of spirometry with a narrow margin, although those were statistically significant.

C5. Discussion
p13 line 154 typo hyperferritinemia

R5.

We apologize for our carelessness and the lack of clarity Thank you for pointing our typo out.

We changed “hyperfrritinemia” into “hyperferritinemia” in discussion section **(from line 348 on page 24 )**.

**References**

**1.**	West JB (2013) GOLD Executive Summary. Am J Respir Crit Care Med 188: 1366-1367.

**2.**	Choi HS, Park YB, Yoon HK, Lim SY, Kim TH, Park JH, et al. (2019) Validation of Previous Spirometric Reference Equations and New Equations. J Korean Med Sci 34: e304.
